# A data-driven approach to improve coffee drying: Combining environmental sensors and chemical analysis

**DOI:** 10.1371/journal.pone.0296526

**Published:** 2024-02-07

**Authors:** Prasara Jakkaew, Yodying Yingchutrakul, Nattapol Aunsri

**Affiliations:** 1 School of Information Technology, Mae Fah Luang University, Chiang Rai, Thailand; 2 National Center for Genetic Engineering and Biotechnology, National Science and Technology Development Agency (NSTDA), Pathum Thani, Thailand; 3 Integrated AgriTech Ecosystem Research Group, Mae Fah Luang University, Chiang Rai, Thailand; West Virginia State University, UNITED STATES

## Abstract

The study introduces a methodology that utilizes data-driven approaches to optimize coffee drying operations. This is achieved through the integration of ambient sensor data and chemical analysis. This statement underscores the significance of temperature regulation, humidity levels, and light intensity within the context of coffee production. There exists a positive correlation between elevated temperatures and increased rates of drying, but humidity has a role in determining the duration of the drying process and the preservation of aromatic compounds. The significance of light intensity in dry processing is also crucial, since excessive exposure can compromise both the taste and quality of the product. The findings of chemical investigations demonstrate a correlation between environmental factors and the composition of coffee. Specifically, increased temperatures are associated with higher quantities of caffeine, while the concentration of chlorogenic acid is influenced by humidity levels. The research additionally underscores the variations in sensory characteristics among various processing techniques, underscoring the significance of procedure choice in attaining desirable taste profiles. The integration of weather monitoring, chemical analysis, and sensory assessments is a robust approach to augmenting quality control within the coffee sector, thereby facilitating the provision of great coffee products to discerning consumers.

## Introduction

In recent years, there has been a growing body of research examining the phytochemical composition of coffee and its potential health benefits. Specifically, some studies have shown that regular coffee drinking may be associated with a decreased risk of developing type 2 diabetes [1], colorectal cancer [2, 3], and cardiovascular illnesses [4, 5]. In recent times, there has been a significant increase in global consumer interest in coffee, mostly due to its perceived health advantages, distinctive scent, and unique bitterness. The coffee industry is constantly seeking ways to enhance the quality and consistency of coffee production [6]. One critical aspect of coffee production is the drying process [7–10], which plays a pivotal role in determining the final quality and flavor of the coffee beans [11]. The drying process involves reducing the moisture content of harvested coffee cherries to an optimal level that preserves the desired flavors, aromas, and chemical composition [12, 13]. Proper drying conditions are essential to ensure the development of desirable chemical compounds while preventing the growth of undesirable microorganisms. Variations in drying conditions can lead to inconsistent coffee quality and impact the flavor, aroma, and overall cup profile [14, 15]. Therefore, optimizing the drying process is crucial to ensure a superior coffee product. Weather conditions, such as temperature, humidity, and light intensity, significantly impact the drying rate and quality of coffee beans [11, 16–18]. Monitoring these environmental factors is crucial for coffee producers to make informed decisions and ensure optimal drying conditions [19]. The interaction among these elements is crucial in determining the chemical composition and sensory characteristics of the coffee beans [15, 20]. The monitoring and management of these factors play a crucial role in promoting consistent quality and improving the entire coffee manufacturing process. However, the huge amount of data derived by sensors integrated into coffee drying facilities presents a challenge in terms of effective analysis and interpretation. The sheer volume and intricacy of data produced by these sensors might potentially inundate conventional methods of data processing. Coffee growers encounter the task of deriving significant insights from the abundant data supplied by sensors in order to make well-informed choices.

In recent years, the integration of advanced technologies, particularly the use of environmental sensors and chemical analysis [21–23], has revolutionized agricultural practices. By employing data-driven approaches, it is now possible to monitor and control environmental conditions with precision, allowing for a more nuanced and efficient drying process. This convergence of technology and agriculture holds great promise for enhancing the overall quality and consistency of coffee production. Moreover, this study explores the connection between the chemical constituents present in coffee beans and the sensory data collected throughout the drying process. The quantities of chemical compounds such as caffeine, theophylline, and chlorogenic acid are being carefully examined, with a particular focus on their interactions with environmental factors. Revealing these relationships enhances our understanding of the elements that influence the quality and taste of coffee.

The primary objective of this study is to investigate the feasibility of integrating atmosphere sensor data with chemical analysis techniques in the context of coffee drying. This study measures the influence of environmental elements on the coffee production process, examines the chemical composition of coffee beans, establishes correlations between the environment and chemical components, and evaluates the association between data-driven optimization techniques and coffee cupping scores. The objective of this study is to offer a comprehensive analysis of the potential benefits that data-driven methodologies might bring to the coffee drying process, ultimately resulting in enhanced coffee quality during production.

### Coffee drying process and its impact on coffee quality

The coffee drying process is a crucial stage in coffee production, significantly affecting the final quality of the beans [12, 19]. Controlling drying parameters, such as temperature, humidity, and drying time, leads to consistent and high-quality coffee beans [12, 24, 25]. Various methods are employed for coffee drying, encompassing natural (sun) drying, mechanical drying, and solar drying. Sun drying, prevalent due to its cost-effectiveness, exposes coffee beans to sunlight, fostering distinctive flavors [26]. Nonetheless, its efficacy hinges on climate conditions, making it time-intensive. The duration of sun drying varies depending on the coffee type, ranging from 7-15 days for parchment coffee, 12-21 days for parchment coffee with mucilage, to 30-45 days for drying cherry coffee [26]. Despite its advantages, sun drying is labor-intensive, susceptible to weather variations, and exposed to potential risks such as dust and water pollutants compromising bean quality. In contrast, mechanical and solar drying is swifter but fail to replicate the flavor profile of sun-dried coffee. Mechanical drying offers faster results and better process control but is accompanied by heightened costs and the peril of over-drying [27]. Solar drying, conversely, taps into solar heat, fostering environmental sustainability due to the absence of fuel or energy consumption [28].

To ensure effective drying, several factors must be carefully managed. Temperature plays a significant role, as a higher temperature difference between the heating medium and the coffee tissue enhances heat transfer and moisture evaporation. It is recommended to maintain an air temperature between 49°C and 60°C during the drying process, gradually reducing it to less than 55°C towards the end [29]. Relative humidity is crucial, as lower humidity enables the air to absorb more moisture. Adequate ventilation is essential to prevent mold and ensure proper airflow. The surface area of the coffee beans impacts water vapor removal, and controlling light exposure is necessary to avoid overheating and flavor degradation [30]. Thus, meticulous control over temperature, humidity, airflow, light exposure, and surface area is imperative for consistent and high-quality coffee beans [25].

### Role of sensor data in analyzing coffee quality

Sensor data is crucial in analyzing coffee quality, offering objective insights into factors influencing the final product outcome. In the coffee drying process, sensors like temperature, humidity, and light intensity are strategically deployed for environmental monitoring [31]. These collected data offer insights into the conditions experienced by coffee beans during drying, allowing for the assessment of the relationship between these conditions and coffee quality attributes. Existing weather monitoring systems for coffee drying have been developed and implemented to optimize the process and ensure consistent quality of the coffee beans [32]. These systems utilize sensors strategically placed to measure and monitor key environmental factors that influence the drying process, such as temperature, humidity, light intensity, and wind speed [32].

The sensors integrated into these systems provide real-time data on the environmental conditions during the coffee drying process, which is collected and transmitted to a central control unit or connected device for analysis and decision-making. By having access to accurate and up-to-date information, coffee producers can make informed decisions regarding the management and adjustment of drying conditions to achieve optimal results [32]. One common approach in these systems is the use of microcontrollers, such as Arduino or Raspberry Pi, which act as the central control unit. These microcontrollers can be equipped with connectivity options like Wi-Fi or GSM to enable remote monitoring and analysis of the data.

The implementation of weather monitoring systems for coffee drying has shown promising results in improving the overall efficiency and quality of the process. By closely monitoring and controlling environmental factors, coffee producers can optimize the drying conditions to achieve consistent and desirable outcomes. For example, adjusting temperature to prevent over-drying or flavor degradation, or increasing humidity to avoid excessive moisture loss from the beans, these systems enables coffee producers to gather valuable data over time, facilitating analysis and identification of patterns or correlations between environmental conditions and resulting coffee quality. This information can be used to refine and optimize the drying process in the long term, leading to improved consistency and quality in coffee production.

### Data driven approach in agricultural

The agricultural industry is undergoing a significant transformation due to the adoption of data-driven techniques. These methodologies are playing a crucial role in improving the sustainability of food systems. This is achieved by several means, such as the automation of processes, the utilization of large-scale datasets, the enhancement of human-computer interactions [33], and the curation of data [34]. The processing of big data entails the utilization of machine learning and deep learning methodologies, whereas automation encompasses the integration of sensors, robotic systems, and earth observation satellite systems. The advent of artificial intelligence (AI) has brought about a significant transformation in various scientific fields [35], facilitating enhanced decision-making capabilities. Nevertheless, the intricate nature of agricultural data presents significant obstacles. The acquisition of precise and reliable data is of utmost importance in the development of models aimed at predicting trends and examining the effects of climate change on agricultural productivity. The utilization of data-driven methodologies is revolutionizing contemporary agricultural practices through the enhancement of resource allocation, accurate prediction of crop yields, and increased resilience to climate unpredictability. Precision agricultural approaches facilitate farmers in making accurate modifications to irrigation schedules, insect control, and fertilizer use, thereby boosting crop output. The primary objective of technological improvements is to augment climate resilience and alleviate associated dangers. The primary emphasis of collaborative endeavors lies in the development and implementation of crop varieties that possess enhanced resistance to drought conditions, the utilization of automated systems for harvesting purposes, and the exploration of alternative agricultural methodologies.

## Methodology

This study used a four-stage approach to comprehensively examine the correlation between environmental factors and the chemical composition during the process of coffee drying. The initial phase entails the selection of environmental sensors for the measurement of temperature, humidity, and light intensity. The subsequent phase entails the extraction and subsequent analysis of chemical constituents, including but not limited to caffeine, theophylline, and chlorogenic acid. In the third stage, the integration of sensor data with chemical analysis results takes place, wherein correlations are analyzed. The ultimate phase amalgamates environmental and chemical data to facilitate a thorough study, thereby unveiling the intricate interconnections between environmental influences and chemical elements.

The research used an observational research approach to examine sensor data collected throughout the coffee drying process, with a specific emphasis on temperature, humidity, and light intensity. Accurate measurements are captured by carefully placing sensors inside the drying plant. The study was carried out in Mae Ton Luang, Chiang Mai, Thailand, using a site-specific methodology to examine several aspects influencing the quality of coffee and assess different processing procedures. The coffee plantation was granted approval and access by Kantima Tadplevthong, the agriculturalist and owner of the property. The operations were carried out in accordance with both local regulations and ethical standards. The granting of this authorization facilitated the acquisition of vital data pertaining to temperature, humidity, light intensity, and other characteristics of utmost significance for our inquiry into the coffee drying procedure.

### Sensor selection and data collection

In this phase, a careful protocol was implemented to systematically choose weather sensors with the specific goal of monitoring the progression of the coffee drying procedure. The essential elements of the environment, such as temperature, humidity, light intensity, and wind speed, were comprehensively considered. A thorough assessment was conducted to evaluate the precision, dependability, and interoperability of commercially accessible sensors with the monitoring system. The array of sensors that were chosen included the SHT31 temperature and humidity sensor, an ambient light sensor, and, optionally, an anemometer for the purpose of monitoring wind speed. The sensors were carefully positioned within the confines of the drying region, ensuring comprehensive data collection.

The integration of these sensors was accompanied by a microcontroller, which functioned as the central unit for the acquisition of data and subsequent analysis. A software program was systematically created and implemented with the aim of improving the efficiency of data retrieval. The program incorporated important software libraries specifically tailored to fulfill the distinct demands of the sensors, hence guaranteeing seamless compatibility and exceptional performance. The meticulous arrangement of sensor pins and fine-tuning of settings were performed to enhance the precision of data acquisition.

**Fig 1:**
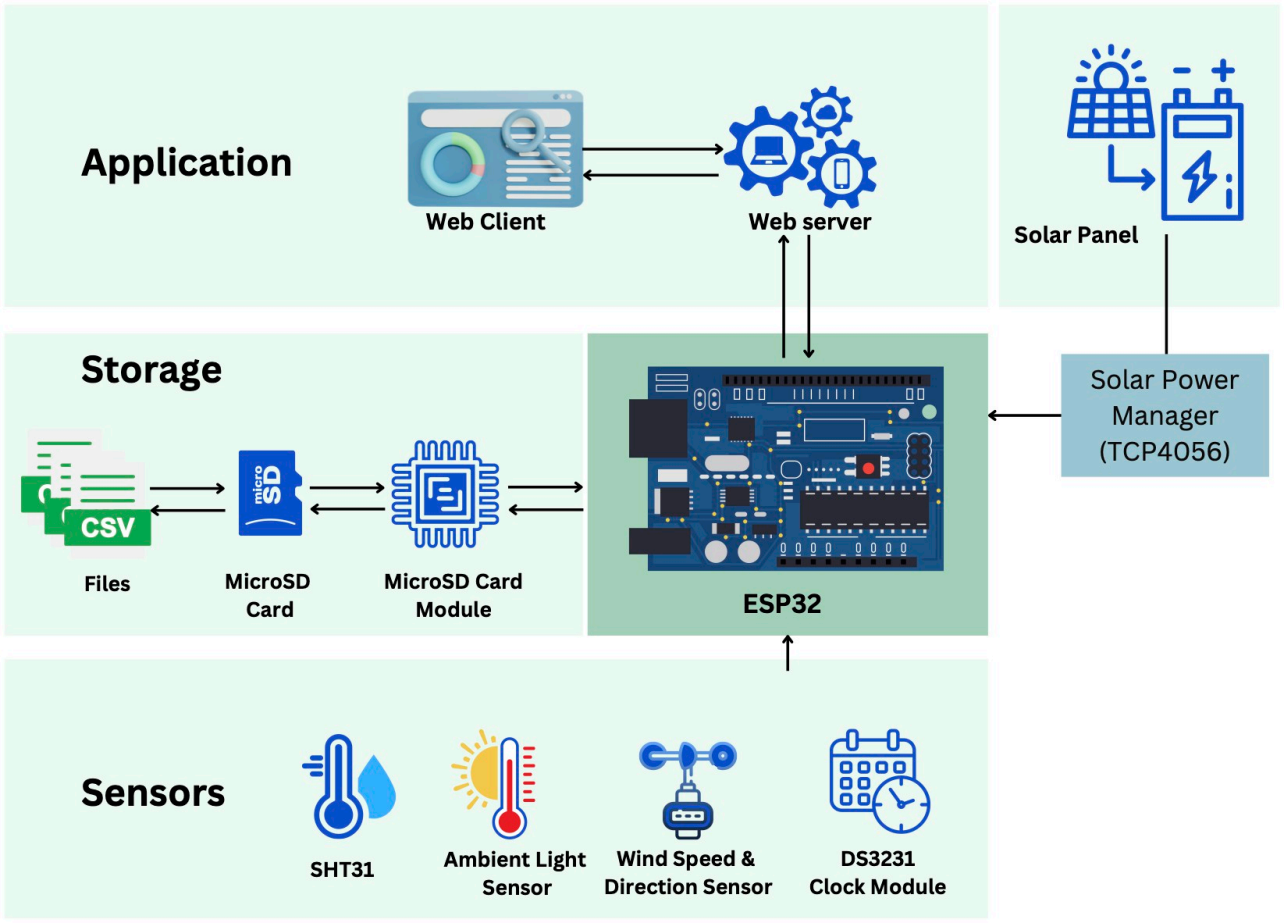
Overview of system architecture.

The weather station’s design demonstrates a unique characteristic as it was specifically engineered to function effectively in remote areas that lack internet connectivity or conventional power sources (see Fig 1). The accomplishment was attained with the incorporation of a WiFi module, facilitating the wireless transmission of data from the microcontroller to a dedicated SD card for the purpose of storage. The utilization of this innovative methodology not only ensured the systematic collection of data in geographically distant regions but also underscored the station’s capacity to endure unfavorable conditions. Furthermore, the integration of solar energy not only enhanced the station’s ability to sustain itself and withstand external factors but also empowered it to operate independently in accordance with environmentally conscious principles.

The research gathered empirical data pertaining to temperature, humidity, light intensity, and wind speed during the coffee drying process. The frequency of the data sample was established at 36 seconds, facilitating the acquisition of consistent readings from the sensors. This facilitated the assessment of the dynamic attributes of the drying environment, including fluctuations in temperature, humidity, light intensity, and wind speed. The period of data collecting spanned a continuous sequence of days, commencing on December 5, 2021. The data-gathering methodology was consistently maintained until a sufficient quantity of information was obtained, enabling the analysis and development of substantial findings about the process of coffee drying. The data presented in this study encompasses a typical sample, which includes measurements of timestamps, temperature, humidity, light intensity, and wind speed. Sensor data was gathered at regular intervals, enabling a thorough comprehension of the coffee drying process.

### Extraction and analysis of chemical components

To explore the correlation between sensor data and chemical components of coffee, such as caffeine, theophylline, and chlorogenic acid, additional chemical analyses will be conducted. Coffee samples will be collected during the drying process, and the desired chemical components will be extracted and analyzed using appropriate laboratory techniques. The obtained chemical data will then be integrated with the sensor data to perform statistical analysis and identify any relationships or dependencies.

One gram of roasted coffee beans prepared using the same cupping profile as the sample, was ground and mixed with 10 mL of methanol in a shaker at 150 rpm for 6 hours at 8°C. The mixture was then centrifuged at 14,000 × g for 30 minutes. Reverse phase-solid Phase Extraction (SPE) with the extraction manifold system was employed to clean up the samples and remove undesirable compounds. The reverse phase-SPE was conditioned using 25 mL of acetonitrile and equilibrated with 50 mL of deionized water (DI). The extracted coffee bean samples were loaded onto the equilibrated SPE and eluted with 100% acetonitrile. The eluates were subsequently evaporated under vacuum using rotary evaporation. The samples were reconstituted in 200 *μ*L of methanol and diluted with 1800 *μ*L of 1% formic acid/water at a 1:10 ratio (v/v) before being subjected to LC-MS/MS analysis. To confirm the reproducibility of the data, quality control of the different extracts was conducted. The external standard compound (caffeine) was utilized to determine the consistency of all independent extraction batches (n = x). Metabolite profiling was analyzed using a Q-Exactive HF Orbitrap Mass Spectrometer coupled with an UltiMate 3000 LC system. The analysis employed a Hypersil GOLD^™^ column held at 50°C.A total of 2 *μ*g (2 *μ*L) sample injections (concentration = 1 *μ*g/*μ*L) were used at a flow rate of 0.3 mL/min. The mobile phase consisted of 90% methanol/water with 0.1% formic acid (MP: A) and acetonitrile with 0.1% formic acid (MP: B) (LC-MS grade, Sigma). The gradient starting conditions were 99% MP:A and 1% MP:B. These conditions were maintained for 0.5 minutes before increasing to 55% B over 20 minutes. The column was flushed with 99% B for 5 minutes before returning to the starting conditions. The total analysis time for each run was 35 minutes. The mass spectrometer was operated in positive mode with a spray voltage of 3.8 kV. The sheath gas and auxiliary gas flow rates were set at 48 and 11 arbitrary units (AU), respectively. The capillary temperature was set at 350°C. The MS analysis alternated between full scans and data-dependent MS/MS scans with dynamic exclusion. For full MS, the scan range was set at 60–700 m/z, with a resolution of 120,000, an AGC target of 3e^6^, and a maximum injection time (max IT) of 100 ms. For full MS/MS, the resolution was set at 30,000, with an AGC target of 1e^5^ and a max IT of 200 ms. Up to six ions (Top6) with the most intense signals were fragmented. To prevent sample contamination, a blank sample (0.1% formic acid/water) was injected after every analysis. All LC-MS runs were acquired using the Xcalibur 3.1 software (Thermo Scientific). The total ion current (TIC) profiles of all raw files were extracted using MZmine 2 software [36].

### Data integration and processing

This section provides an overview of the data preparation, cleaning, and merging procedures employed in conducting a detailed analysis of the coffee drying process. The procedure consists of two main stages: Data Cleaning and Preprocessing, and the Integration of Environmental, Chemical Data and Cupping Analysis data.

#### Data cleaning and preprocessing

The process of data cleaning and preprocessing is an essential and fundamental stage in the preparation of a dataset for analysis or modeling. The process involves the identification and adjustment of errors, the management of missing values, and the conversion of data into a format that is appropriate for subsequent analysis. In the present scenario, the dataset under consideration comprises of time-series data, encompassing various columns such as Timestamp, Temperature, Humidity, and Light Intensity.

To ensure the quality and reliability of the collected sensor data, preprocessing and cleaning techniques are essential. One common technique is to remove rows that contain missing or NaN (Not a Number) values. By removing these rows, we can eliminate any incomplete or unreliable data points, ensuring a more accurate dataset.

In the provided code snippet, the variable data represents the collected sensor data. The method dropna() is applied to the data to remove rows that contain NaN values. The resulting dataset, denoted as data_without_nan, will only include rows with complete and valid data.

By removing rows with NaN values, we can improve the quality and integrity of the dataset, ensuring that subsequent analyses and visualizations are based on reliable and complete data. This preprocessing step is important to obtain accurate and meaningful insights from the sensor data collected during the coffee drying process.

The process of normalizing or standardizing data is performed by applying certain algorithms, whereas categorical variables can be converted into numerical representations by techniques such as immediate encoding. The identification and management of outliers is crucial in statistical analysis. This can be achieved by removing these outliers from the dataset or applying data transformation techniques to reduce their impact on the analysis.

#### Integration of environmental and chemical data

Integration of environmental and chemistry data is a key step in figuring out how the conditions of the drying process affect the chemicals in coffee beans. This part explains how and why these datasets were put together to make a more complete analysis.

**Fig 2:**
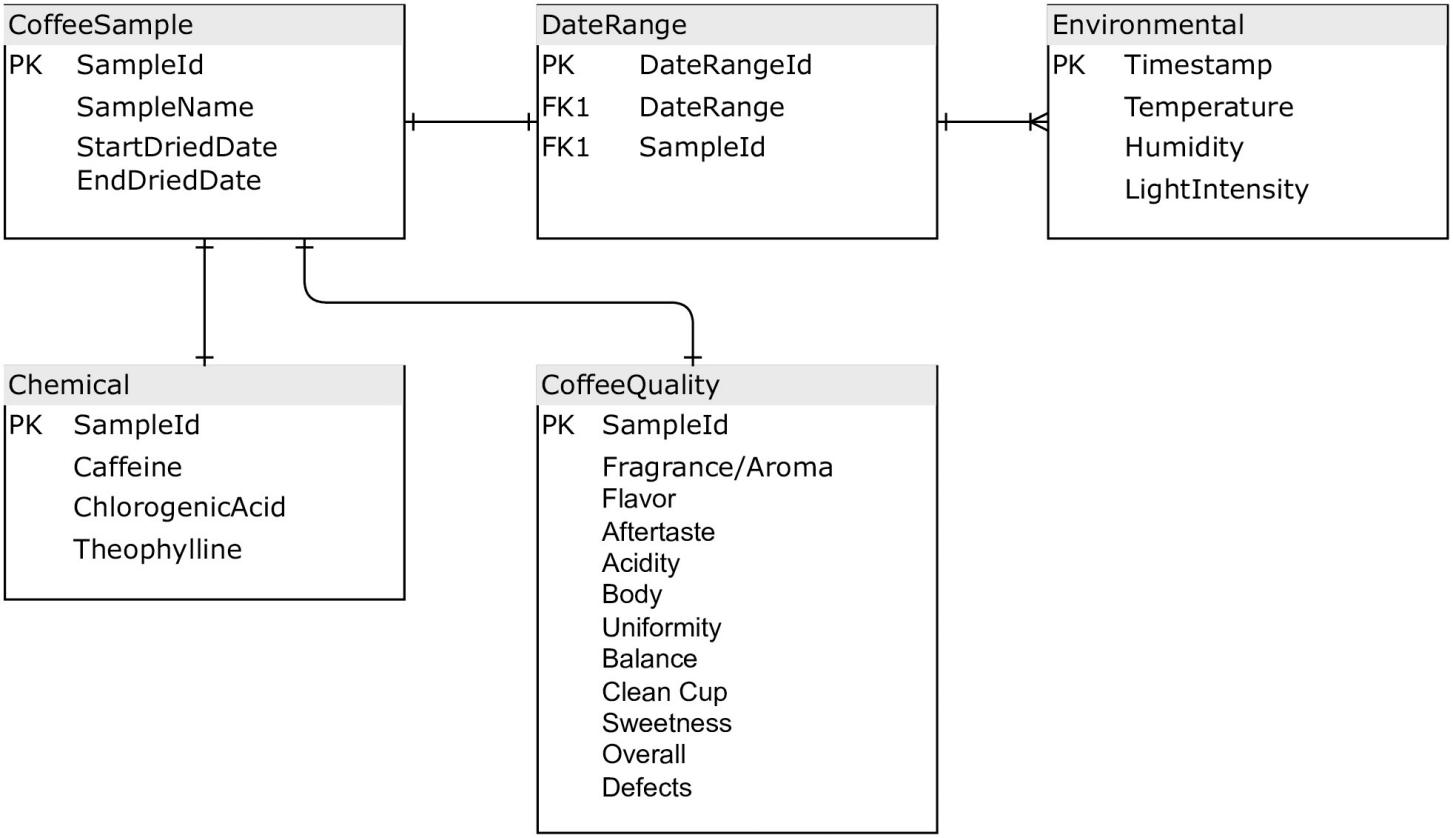
Data relationship ER diagram.

The dataset comprises a time-series data, consisting of four columns: time, temperature, humidity, and light intensity. The data that has been recorded is denoted in units of degrees Celsius (°C), percentage (%), and lx. Each row inside the collection represents a unique measurement that has been documented at a certain date and time. The ER diagram depicted in Fig 2 depicts the interconnections between the data entities. This sets a one-to-many relationship between the entity DateRange and the entity Environmental, indicating that a single date range can be connected with several pieces of environment data. The foreign key in the Environmental, table, known as DateRangeId, establishes a reference to the DateRange table.

#### Statistical analysis

In order to comprehend the interconnections among various data types in our research, a thorough examination was carried out on temperature, humidity, and light intensity inside the sampled area. The summary statistics offer valuable insights into the measures of central tendency, variability, and distribution of the variables under consideration. The initial step involves using descriptive statistics to obtain a comprehensive understanding of the data. These metrics encompass statistical tools such as the mean, median, standard deviation, and correlations. Next, generate visual representations to investigate the connections among variables. An illustration of this concept can be observed using scatter plots, which depict the relationship between composition and several environmental factors such as temperature, humidity, and drying period.

Correlation analysis is employed as a method for assessing the magnitude and orientation of linear associations among variables. The current research utilized a distinct approach to examine the connections among different environmental factors, specifically temperature, humidity, and light intensity, and significant attributes of coffee, such as caffeine concentration, chlorogenic acid levels, and sensory characteristics. The aim of this study was to uncover potential interrelationships that could impact the coffee production process. The utilization of descriptive statistics offered a concise summary of the dataset. The study encompassed the computation of essential statistical measures, such as the arithmetic mean, median, and standard deviation. The summary statistics provided meaningful insights into the central tendencies and dispersion of the observed variables, enabling a comprehensive comprehension of the fundamental attributes of the dataset. The proposed methodology will be employed to ascertain and evaluate the relationships between the environmental factors, acting as independent variables, and the attributes of coffee, which are regarded as dependent variables. The present study aims to construct a conceptual framework that facilitates comprehension of the potential consequences of alterations in environmental conditions on the process of coffee production. Data visualization, while not formally categorized as a statistical test, played a vital role in the analytical process. Visual representations, such as the provided figures, were utilized to elucidate the inherent patterns, trends, and connections present within the data. The incorporation of visual depictions offered a lucid and readily comprehensible approach for conveying complex data, hence enhancing the understanding and evaluation of results.

The application of diverse statistical tests and procedures facilitated a thorough analysis of the data, resulting in a deeper understanding of the correlation between environmental conditions and the characteristics of coffee throughout its production processes. The implementation of a rigorous methodology in this study bolstered the robustness and reliability of the results.

## Results

This study relies on the examination of the coffee production process, employing a dataset that encompasses the time period from December 5, 2021, to March 30, 2022. The dataset comprises a sequential collection of observations, with each observation representing an isolated record of environmental and coffee-related variables such as time, temperature, humidity, light intensity chemical components and cupping score.

### Environmental factors impact on coffee drying

This section provides an analysis of the influence of environmental variables on the process of coffee drying. Specifically, it investigates the effects of temperature, humidity, and light intensity on the pace at which coffee beans undergo drying (see Table 1). The research offers valuable insights into the optimization of the drying process and the attainment of consistent coffee quality.

**Table 1:** Description of samples.

Samples	Dried duration (days)	Mean Temperature (°C)	Mean Humidity (%)	Mean Light Intensity (lx)
D-1	29	17.89	82.06	10781.29
D-2	39	19.30	81.31	11366.79
D-3	40	19.50	81.21	11438.34
D-4	35	19.05	81.50	11175.18
H-1	18	15.41	82.13	10426.41
H-2	15	15.89	77.85	11837.14
H-3	13	18.95	80.69	11553.77
H-4	15	21.87	81.18	11710.51
H-5	13	15.67	79.19	11474.37
WA-1	18	16.19	84.93	9652.99
WA-2	12	15.62	86.16	9073.06
WA-3	10	15.66	80.08	11086.28
WB-1	9	15.67	80.12	10956.13
WB-2	12	17.91	80.73	11046.24
WB-3	14	17.73	84.57	9870.11

#### Temperature

The role of temperature is identified as a crucial factor in the coffee drying process. Higher temperatures facilitate the process of moisture loss, hence potentially speeding the rate at which drying occurs. Based on our data, a clear association is shown between higher temperatures and enhanced drying efficiency. This association highlights the importance of carefully regulated temperature conditions in order to achieve optimal coffee drying efficiency.

**Fig 3:**
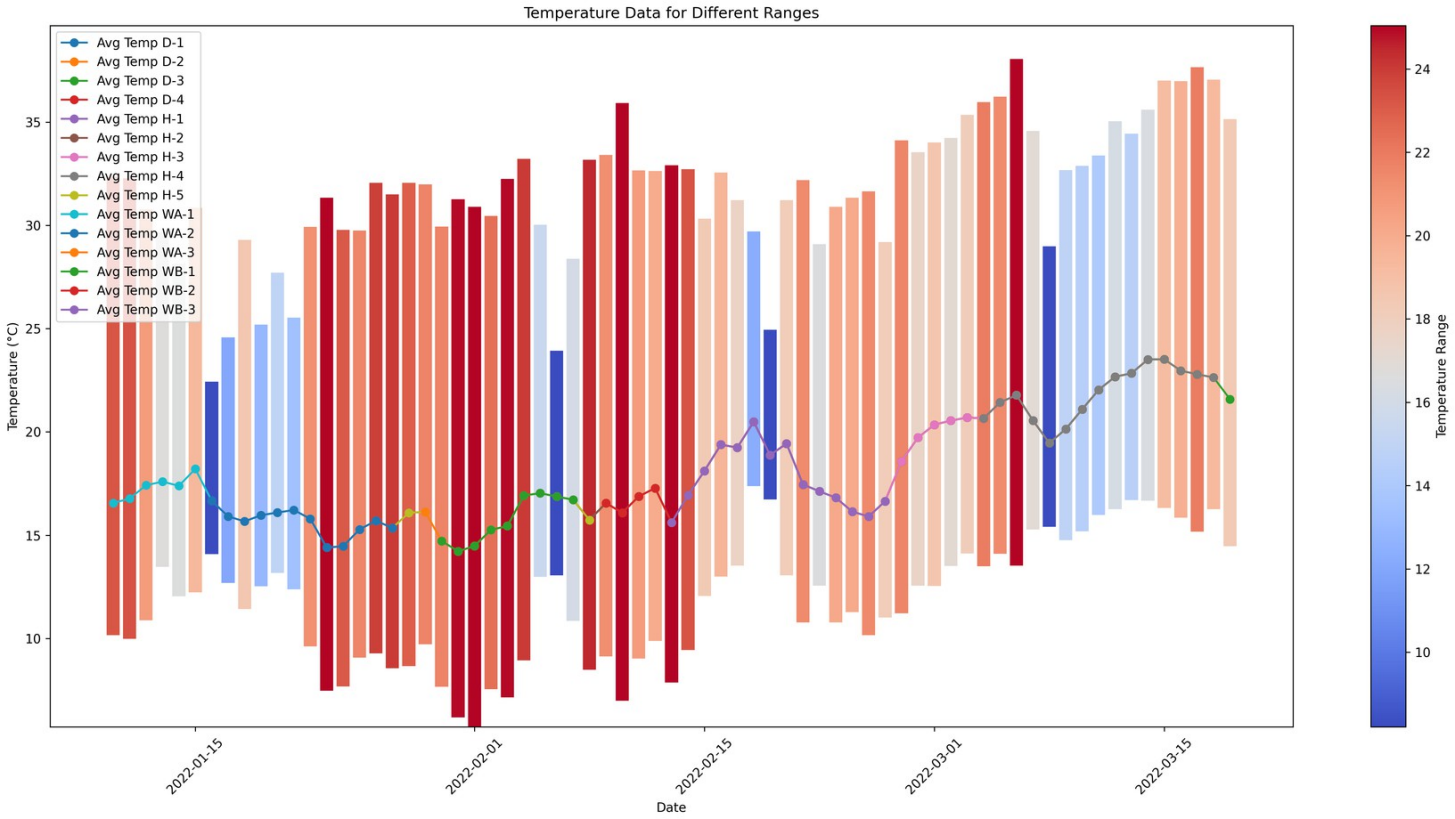
Temperature during drying process.

The temperature throughout the drying process is depicted in the plot utilizing a bar chart (refer to Fig 3). The height of each bar represents the range of temperatures that were measured on a particular day. The coloration of the bars is contingent upon the temperature range, wherein cooler hues denote a more limited range, while warmer hues signify a broader range. To clarify, it might be stated that shades of blue are indicative of relatively little temperature shifts, but warmer hues such as red are associated with more significant temperature fluctuations. Moreover, the bars originate from the minimum recorded temperature, so visually illustrating the distribution of temperatures during the designated timeframe. The presented picture facilitates a comprehensive comprehension of the temporal variations in temperatures during the drying procedure.

The temperature during the coffee drying process is of paramount importance as it significantly influences the flavor, quality, and overall attributes of the end product. The drying period for sample D-3 is extended to 40 days when the temperature ranges from 5.72°C to 38.05°C, suggesting that slightly higher temperatures are associated with a longer duration of drying. The flavor profile is also affected by temperature, so examples exhibiting the broadest temperature range showcase intricate flavors such as arid banana, honey, white chocolate, and cedar wood.

#### Humidity

The role of humidity in the drying process of coffee is of utmost importance as it significantly impacts the quality and flavor characteristics of the finished product. Elevated levels of humidity have a decelerating effect on the process of drying, hence influencing the moisture content of the beans and necessitating extended durations for the drying process. The development of flavor is also influenced by humidity, whereby higher levels of humidity have an impact on the creation of specific chemicals. Nevertheless, an excessive level of humidity might provide a potential hazard in terms of the proliferation of mold and mildew, which can have adverse effects on both the overall quality and safety of the end product.

**Fig 4:**
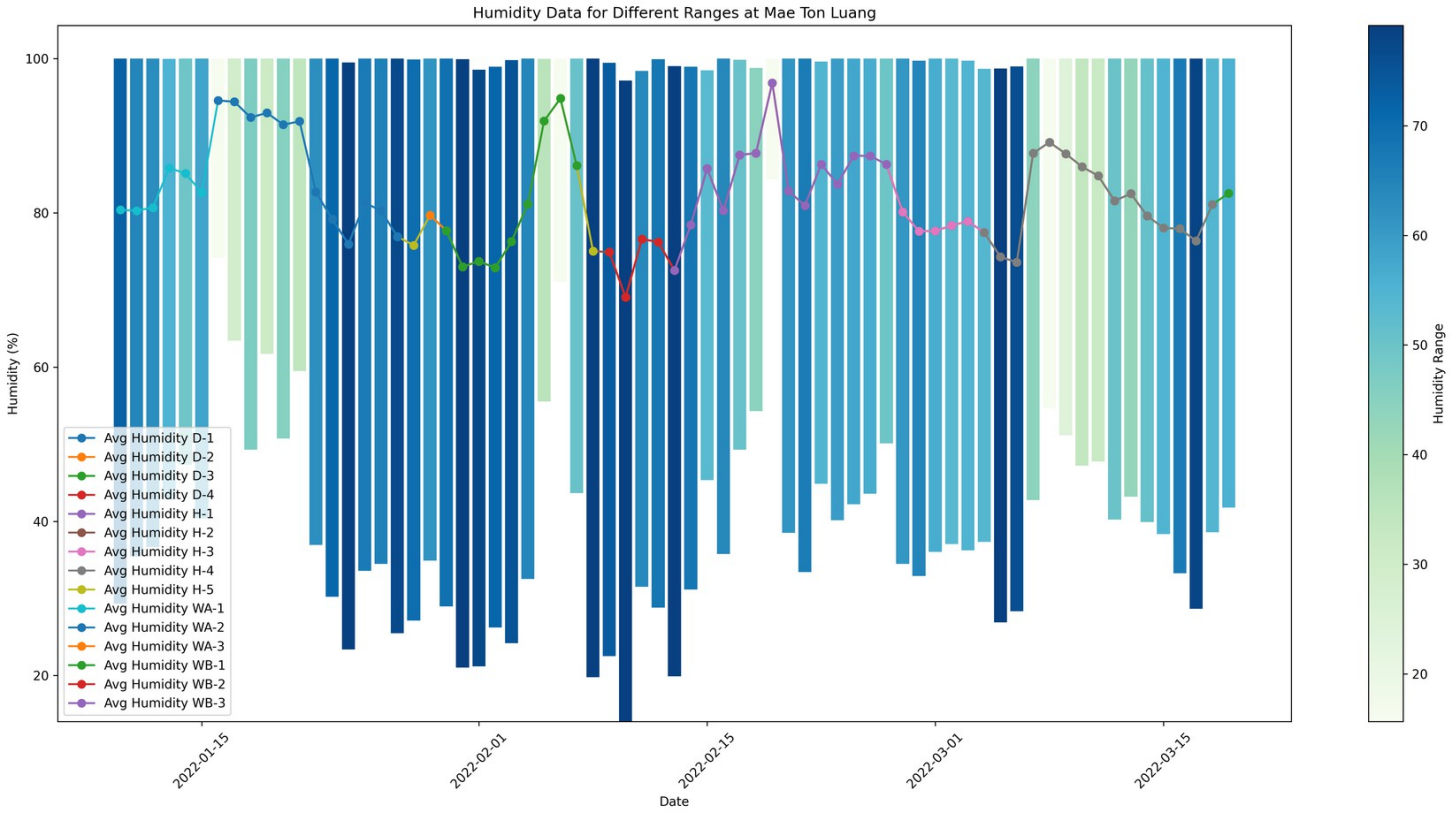
Humidity during drying process.

The graph (Fig 4) shows the humidity levels during the drying process using a bar graph. Each bar represents a different day, with lighter hues indicating a limited range of humidity and darker hues indicating a greater range. The bars originate from the minimum recorded relative humidity value, illustrating the distribution of humidity within the specified period. This concise and inclusive representation effectively communicates the variations in humidity levels observed during the drying process.

The maintenance of appropriate humidity levels is crucial in achieving consistent drying throughout all beans, hence mitigating the occurrence of uneven drying and fluctuations in flavor and overall quality. Furthermore, humidity levels have a significant impact on the retention of aromatic chemicals within coffee beans. Specifically, higher levels of humidity result in a more prominent aroma and fragrance in the final coffee product. Coffee growers are required to adopt environmental control strategies, including the implementation of climate control infrastructure and the utilization of natural drying techniques that take into account the specific humidity levels of the local environment. A comprehensive comprehension of the fluctuations in humidity levels across different seasons is necessary in order to uphold a consistent level of quality. Conducting experiments in a controlled setting to manipulate humidity levels can facilitate the determination of the ideal range for specific coffee kinds and processing techniques. In essence, humidity plays a pivotal role in the process of coffee drying, exerting a significant influence on the ultimate quality, flavor, and overall attributes of the end product. Monitoring and controlling humidity levels during the drying process is crucial for producers in order to attain desired flavor profiles and uphold product uniformity.

High overnight humidity, as detected by our experiment’s weather monitoring system in Northern Thailand, which could affect coffee drying. When drying coffee beans, farmers should flip them frequently to keep the coffee evenly thick and covered during periods of high humidity. Humidity problems can be overcome and sustainable habits can be spread with the use of locally adapted instruments including adaptable mechanical dryers, solar dryers, hybrid drying systems, and humidity-controlled facilities. It is essential for these strategies to be advanced through local innovation and education. Strategically putting these plans into action guarantees constant quality and gives people the tools they need to improve drying procedures regardless of weather. Taking these steps can improve coffee quality and resistance to high humidity levels.

#### Light intensity

**Fig 5:**
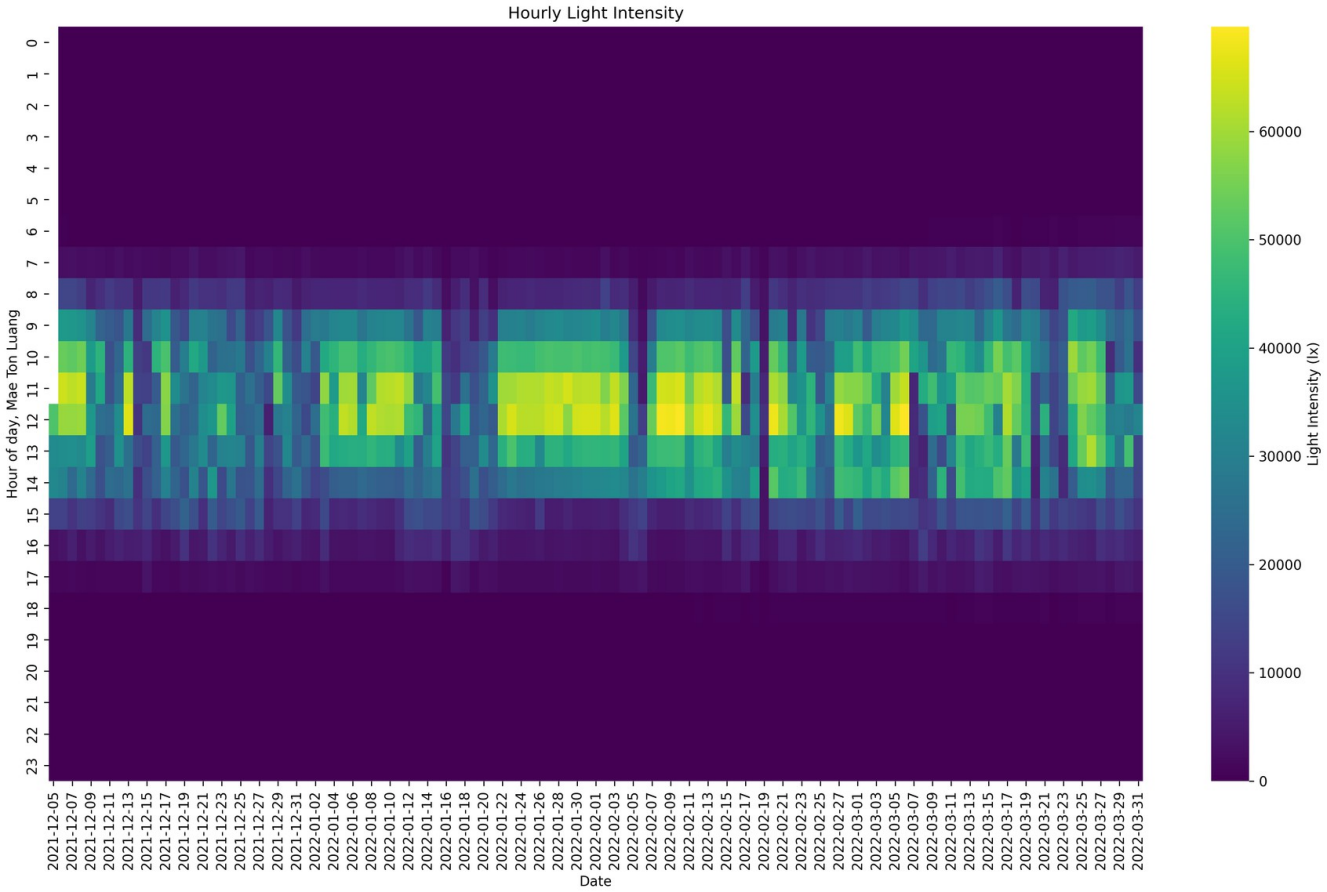
Light intensity during drying process.

The coffee drying process is notably influenced by the average light intensity (Fig 5), which has a substantial impact on various aspects such as drying efficiency, taste profiles, and the overall quality of the cup. Dry processing methods, namely Dry, Honey, and Washed, indicate a tendency towards increased average light intensity, perhaps leading to enhanced drying efficiency in areas characterized by elevated levels of humidity. Implementing this measure can effectively inhibit the growth of mold and successfully get the targeted level of moisture content.

The flavor and fragrance characteristics of coffee exhibit variation across distinct processing methods, wherein heightened light intensity has been observed to exert an influence on the sensory attributes of taste and aroma. The dryness of processed samples can be attributed to the elevated average light intensity, which may lead to a reduction in moisture content inside the green beans. Producers ought to exercise caution over excessive drying, particularly in areas characterized by high solar radiation, as extended exposure may result in excessive drying and have a detrimental impact on the taste and overall quality of the coffee. Achieving a uniform drying process is of utmost importance, as it ensures an even distribution of light throughout the drying process. Any deviation from this uniformity can result in inconsistent flavor profiles.

One potential area for development involves reassessing the drying circumstances, such as light exposure, in order to enhance the overall quality of the cup. Environmental factors should be given due attention, including the utilization of natural sunshine and the adoption of energy-efficient lighting systems. In essence, the coffee drying process is significantly influenced by the average light intensity, necessitating producers to effectively regulate light exposure in order to get desired flavor profiles and ensure high quality.

### Chemical analysis

The chemical analysis of caffeine, theophylline, and chlorogenic acid in the coffee samples was conducted to examine their levels and establish correlations with the corresponding weather conditions. Statistical analysis was performed to identify significant relationships between specific weather variables, including temperature, humidity, light intensity, and wind speed, and the concentrations of these key compounds. The findings revealed noteworthy associations between weather conditions and the chemical composition of coffee.

Higher temperatures were found to be positively correlated with increased caffeine content in the coffee samples. This suggests that elevated temperatures during the coffee drying process may lead to higher caffeine levels. Conversely, humidity levels were observed to influence the concentration of chlorogenic acid in the coffee. The study demonstrated that higher humidity was associated with higher chlorogenic acid levels in the samples. These correlations between weather conditions and chemical compounds highlight the direct impact of environmental factors on the composition of coffee. The results contribute to a deeper understanding of the relationship between weather conditions, coffee quality, and chemical composition. They underscore the importance of monitoring weather conditions and considering their influence on the chemical profile of coffee. By recognizing the impact of environmental factors, coffee producers can make informed decisions and implement appropriate measures to optimize coffee quality during processing.

However, it is important to note that the correlations observed in this study do not establish a cause-and-effect relationship. Further research is needed to elucidate the underlying mechanisms and establish more precise correlations between weather conditions and coffee chemical composition. Additionally, considering other factors such as soil composition, altitude, and agricultural practices could provide a more comprehensive understanding of the complexities involved in coffee production.

The integration of weather monitoring, traceability systems, sensory evaluations, and chemical analysis can collectively contribute to enhancing coffee quality control and decision-making processes in the coffee industry. By leveraging these comprehensive approaches, coffee producers can gain valuable insights into the influence of weather conditions on coffee quality, make informed decisions during processing, and ultimately deliver exceptional coffee products to consumers. Table 2 provides the relationship between environmental parameters and caffeine, chlorogenic acid, and theophylline levels in coffee samples. The coffee samples were categorized based on the processing method: washed, honey, and dry. The environmental parameters were the dried duration, average temperature, average humidity, and average light exposure.

**Table 2:** Environment parameters and compound levels in coffee samples.

Process	Dried duration (days)	Average Temp	Average Hum	Average Light	Caffeine (ng)	Chlorogenic Acid (ng)	Theophylline (ng)
Washed	18	16.18	84.45	9812.87	224.11	77.04	0.71
Washed	12	15.66	85.36	9351.87	232.86	79.49	0.67
Washed	10	15.77	80.21	11123.71	362.55	104.79	1.51
Washed	9	15.74	80.26	10997.33	176.61	49.64	0.47
Washed	12	17.87	80.74	11230.30	229.08	85.74	0.86
Washed	14	17.78	84.27	10145.79	251.02	90.82	0.88
Honey	18	15.46	82.26	10420.81	217.25	90.19	0.81
Honey	15	16.04	78.15	11819.82	246.72	121.48	0.93
Honey	13	19.06	81.19	11176.48	259.18	95.67	0.96
Honey	15	21.85	81.26	11773.70	273.46	82.09	1.30
Honey	13	15.82	79.28	11513.44	249.16	97.36	1.00
Dry	29	18.01	81.80	10898.91	344.52	97.56	1.23
Dry	39	19.38	81.30	11378.80	400.01	123.71	1.99
Dry	40	19.52	81.37	11304.92	244.18	101.94	0.86
Dry	35	19.17	81.41	11238.83	335.41	96.33	1.73

The results showed that the washed coffee samples had a dried duration ranging from 9 to 18 days, with an average temperature between 15.66°C and 17.87°C. The average humidity for the washed samples varied from 80.21% to 85.36%, and the average light exposure ranged from 9351.87 lx to 11230.30 lx. The levels of caffeine in the washed samples ranged from 176.61 ng to 362.55 ng, chlorogenic acid levels ranged from 49.64 ng to 104.79 ng, and theophylline levels ranged from 0.47 ng to 1.51 ng. The dried duration for the honey coffee samples ranged from 13 to 18 days, with an average temperature between 15.46°C and 21.85°C. The average humidity for the honey samples varied from 78.15% to 82.26%, and the average light exposure ranged from 10420.81 lx to 11819.82 lx. The levels of caffeine in the honey samples ranged from 217.25 ng to 273.46 ng, chlorogenic acid levels ranged from 82.09 ng to 121.48 ng, and theophylline levels ranged from 0.81 ng to 1.30 ng. In the case of the dry coffee samples, the dried duration ranged from 29 to 40 days, with an average temperature between 18.01°C and 19.52°C. The average humidity for the dry samples varied from 81.30% to 81.80%, and the average light exposure ranged from 10898.91 lx to 11378.80 lx. The levels of caffeine in the dry samples ranged from 244.18 ng to 400.01 ng, chlorogenic acid levels ranged from 96.33 ng to 123.71 ng, and theophylline levels ranged from 0.86 ng to 1.99 ng.

In general, the outcomes suggest that environmental factors such as drying duration, average temperature, humidity, and light exposure could influence the levels of caffeine, chlorogenic acid, and theophylline in coffee samples. Furthermore, dry processing demonstrated increased variability in theophylline levels, generally presenting slightly higher concentrations than other methods. These findings underscore the substantial impact of coffee processing methods on crucial compounds like caffeine, chlorogenic acid, and theophylline. Specifically, dry processing tended to yield higher levels of caffeine and chlorogenic acid, while theophylline levels displayed greater variability across all methods, indicating distinct variations in compound concentrations influenced by different processing techniques.

### Cup quality analysis

The process of evaluating the quality of coffee cups involves a systematic examination of both the coffee beans and the brewed beverages. This assessment is conducted by an assessment team of three Q Graders who have obtained certification in this field. It includes visual examination, fragrance/aroma evaluation, grind consistency analysis, aroma after grinding, brewing and cupping, taste and sensory assessment, overall impression, defect identification, scoring, documentation and reporting, recommendations for quality improvement, and certification and traceability. The process involves examining coffee beans for uniformity, evaluating fragrance and aroma, assessing grind consistency, and evaluating aroma after grinding. The overall impression of coffee is evaluated, and defects are identified.

The findings of the Cup Quality Analysis are reported in Table 3 illustrates a complete examination of the unique flavor attributes seen in diverse coffee samples subjected to different processing methods, specifically Washed (WA-1, WA-2, WA-3, WB-1, WB-2, WB-3), Honey (H-1, H-2, H-3, H-4, H-5), and Dry (D-1, D-2, D-3, D-4). Every row in the table represents a distinct taste characteristic, such as Fragrance/Aroma, Flavor, Aftertaste, Acidity, Body, Uniformity, Balance, Clean Cup, Sweetness, Overall, and Defects. The columns in the dataset correspond to individual samples categorized according to flavor notes.

**Table 3:** Cup quality analysis results.

**Taste Note/Samples**	**WA-1**	**WA-2**	**WA-3**	**WB-1**	**WB-2**	**WB-3**	**H-1**	**H-2**
Process	Washed	Washed	Washed	Washed	Washed	Washed	Honey	Honey
Fragrance/Aroma	7.00	7.00	6.75	7.00	7.00	7.00	7.00	7.00
Flavor	7.75	7.75	7.50	7.75	7.25	7.50	7.50	7.75
Aftertaste	7.50	7.75	7.25	7.75	7.00	7.25	7.50	7.50
Acidity	7.75	7.50	7.50	7.75	7.00	7.50	7.50	7.50
Body	7.50	7.75	7.75	7.75	7.50	7.75	7.75	7.75
Uniformity	10.00	10.00	10.00	10.00	10.00	10.00	10.00	10.00
Balance	7.50	7.25	7.25	7.75	7.50	7.25	7.50	7.50
Clean Cup	10.00	10.00	10.00	10.00	10.00	10.00	10.00	10.00
Sweetness	10.00	10.00	10.00	10.00	10.00	10.00	10.00	10.00
Overall	7.50	7.50	7.25	7.75	7.50	7.50	7.50	7.50
Defects	0.00	0.00	0.00	0.00	0.00	0.00	0.00	0.00
Totals	82.50	82.50	81.25	83.50	80.75	81.75	82.25	82.50
**Taste Note/Samples**	**H-3**	**H-4**	**H-5**	**D-1**	**D-2**	**D-3**	**D-4**	
Process	Honey	Honey	Honey	Dry	Dry	Dry	Dry	
Fragrance/Aroma	7.25	7.25	7.50	7.75	7.50	7.50	7.25	
Flavor	7.50	7.25	7.50	7.75	7.50	7.75	7.25	
Aftertaste	7.00	7.00	7.50	7.25	7.50	7.50	7.25	
Acidity	7.25	7.25	7.25	7.25	7.25	7.50	7.75	
Body	7.50	7.50	7.25	7.75	7.75	7.75	7.75	
Uniformity	10.00	10.00	10.00	10.00	10.00	10.00	10.00	
Balance	7.25	7.25	7.50	7.25	7.50	7.50	7.25	
Clean Cup	10.00	10.00	10.00	10.00	10.00	10.00	10.00	
Sweetness	10.00	10.00	10.00	10.00	10.00	10.00	10.00	
Overall	7.25	7.25	7.50	7.25	7.50	7.50	7.25	
Defects	0.00	0.00	0.00	0.00	0.00	0.00	0.00	
Totals	81.25	80.75	82.25	81.75	82.75	83.25	81.25	

In the category of Fragrance/Aroma, the ratings vary between 6.75 and 7.75. It is worth noting that the samples WA-1, WA-2, WB-1, H-2, H-3, H-5, D-1, D-2, and D-4 exhibit the highest score of 7.75, suggesting a constant and elevated amount of fragrance/aroma in these particular samples. Likewise, within the Flavor category, the ratings exhibit a range spanning from 7.25 to 7.75. The samples WA-2 and H-1 achieved the highest score of 7.75, signifying the presence of robust and appealing flavors. The Totals row located at the bottom of the table presents the aggregated scores for each processing method. The provided summary reveals that the samples subjected to the Washed method (WA-1, WA-2, WA-3) had a cumulative score of 82.50. In contrast, samples processed using the Honey method (H-1, H-2, H-3, H-4, H-5) and the Dry method (D-1, D-2, D-3, D-4) achieved total scores of 82.25 and 81.25, respectively.

**Fig 6:**
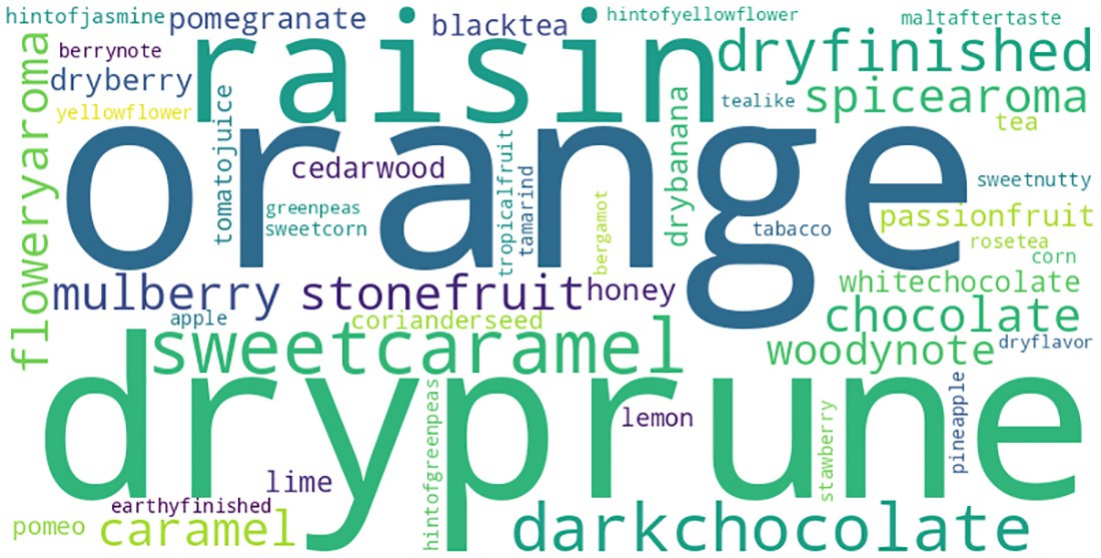
Aroma and flavor notes of all coffee sample.

Fig 6 illustrates a graphical depiction that highlights the prevailing descriptors for flavor and fragrance. In the word cloud, the size of terms is determined by their frequency of occurrence in the dataset. Consequently, terms such as “dark chocolate” or “orange,” which are more frequently referenced in the descriptions of aroma and flavor, will be visually emphasized through higher font sizes. On the other hand, phrases that are less frequently referenced, such as *tealike*or *tabacco*, will be displayed in reduced font sizes. The chosen display approach successfully emphasizes the significance of particular descriptors within the dataset.

### Correlation between environmental conditions and chemical components

**Fig 7:**
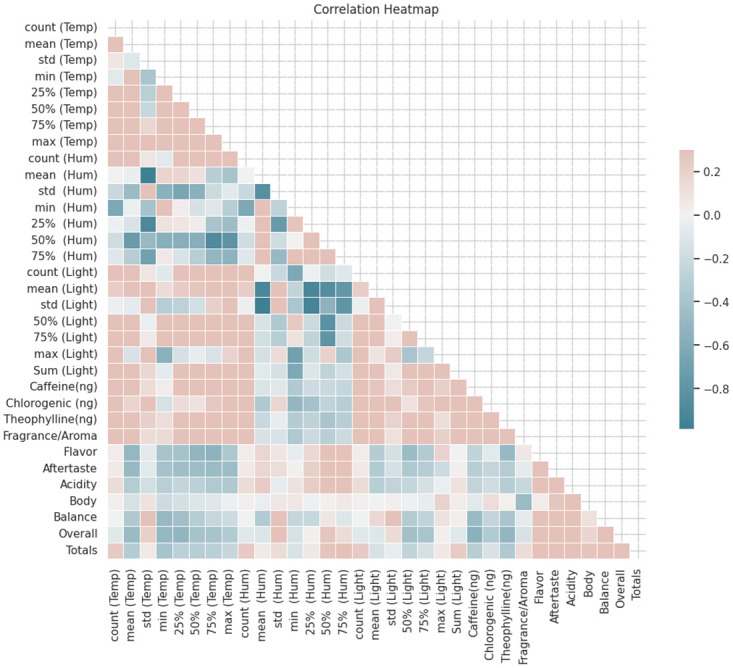
Correlation heatmap.

In order to examine the association between environmental factors (such as temperature, humidity, and light intensity) and chemical properties (including Caffeine, Chlorogenic, and Theophylline) in relation to cup quality analysis, it is possible to perform a correlation analysis using the given correlation matrix (see Fig 7). The primary aim of this research is to investigate the correlations between environmental factors and chemical characteristics, and the examination of cup quality.

The variable under investigation demonstrates moderate positive correlations with Caffeine(ng) (about 0.36), Chlorogenic (ng) (almost 0.19), and Theophylline(ng) (almost 0.48). This discovery suggests a possible association between higher temperatures and greater quantities of these chemicals.

Humidity, alternatively known as Hum, denotes the quantification of the moisture content inside the surrounding atmosphere. A discernible correlation is present between the observed phenomenon and the underlying chemical characteristics, albeit to a restricted extent. The variable that demonstrates the most robust link is Theophylline(ng) with a correlation coefficient of around -0.22. This data suggests a small negative correlation, indicating a potential association between elevated humidity and reduced concentrations of Theophylline. The term “light” pertains to electromagnetic energy found within the visible spectrum, possessing the ability to induce stimulation. The correlations between light and chemical properties often demonstrate a limited degree of relationship. The variable demonstrating the most robust correlation is Fragrance/Aroma, with a coefficient of around 0.23, indicating a modest positive relationship.

Based on the correlation matrix supplied, the links between environmental variables (Temperature, Humidity, Light) and sensory qualities (Fragrance/Aroma, Flavor, Aftertaste, Acidity, Body, Balance, Overall, and Totals) can be observed. The variable being referred to is temperature, commonly abbreviated as temp. There exists a moderate to strong positive link between the variables Flavor and Aftertaste, with correlation coefficients of roughly 0.77 for both. This implies that there may be a positive correlation between elevated temperatures and increased assessments of flavor and aftertaste. Additionally, there exists a moderate positive correlation (about 0.51) between the variable Temperature and the variable Overall. This suggests that an increase in temperature may be associated with higher scores in the overall category.

Humidity, also referred to as Hum, is a measure of the amount of moisture or water vapor present in the air. The variable Flavor exhibits a mild negative correlation, estimated to be roughly -0.50. Similarly, the variable Aftertaste has a slight negative correlation, estimated to be approximately -0.49. This observation implies that an increase in humidity levels could potentially be linked to a decrease in flavor and aftertaste evaluations.

#### Dry process

**Fig 8:**
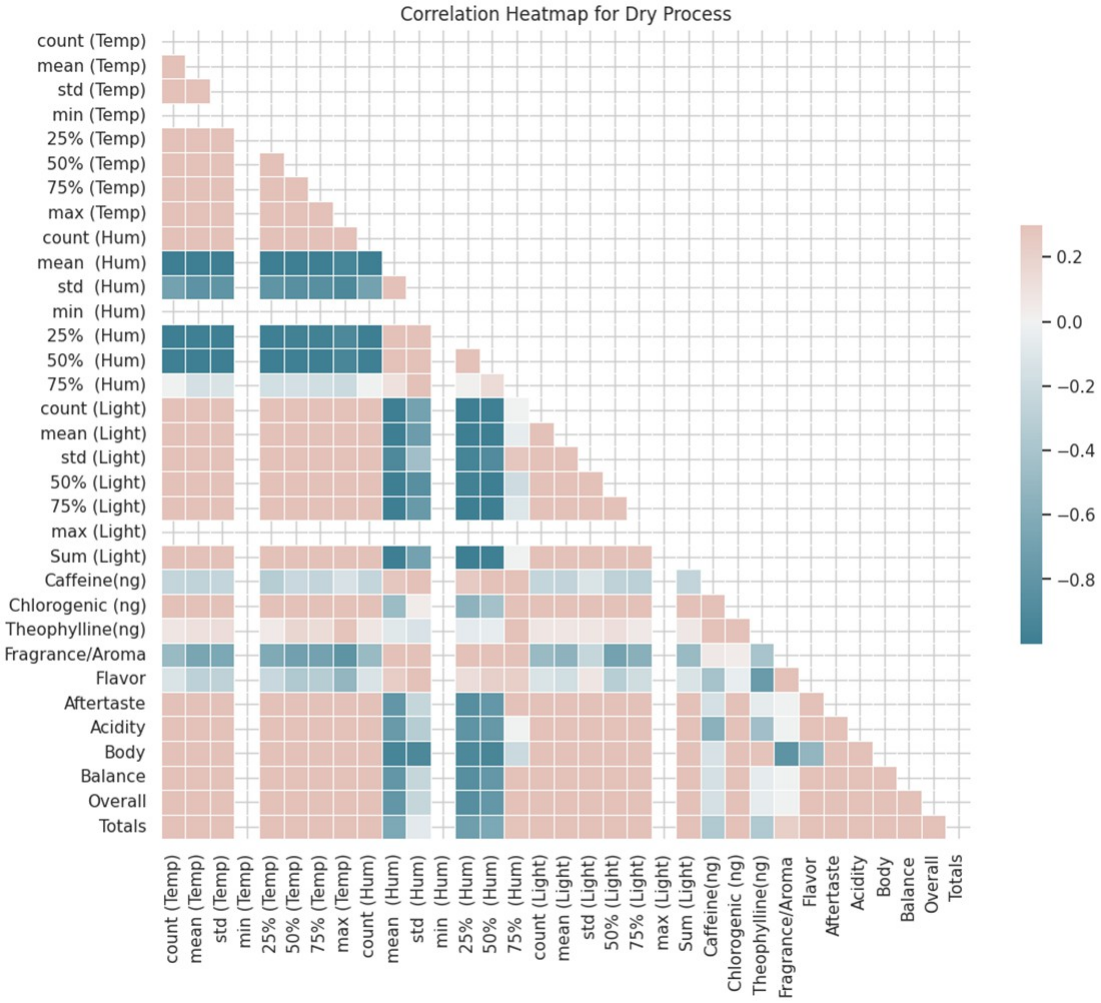
Correlation heatmap for dry process.

The correlation analysis reveals the associations between many environmental elements, namely temperature, humidity, and light, and sensory qualities including fragrance/aroma, flavor, aftertaste, acidity, body, balance, overall perception, and overall quality scores, specifically for the dry process as shown in Fig 8.

*Temperature:* Positive correlations were observed between many aspects of the subject, including Aftertaste (0.87), Acidity (0.82), Body (0.90), Balance (0.87), and Overall (0.87). This implies that elevated temperatures could potentially result in increased scores for these characteristics. Negative correlations were observed between the variables Fragrance/Aroma and Flavor with respective coefficients of -0.49 and -0.12, indicating a moderate negative relationship. This suggests a potential correlation between greater temperatures and lower evaluations for these traits.

*Humidity:* The concept of humidity refers to the amount of moisture present in the air. The qualities of Fragrance/Aroma and Flavor have a strong negative connection, with coefficients of -0.49 and -0.12, respectively. There is a negative correlation between higher humidity levels and lower scores for these qualities.

*Light Intensity:* Positive correlations refer to a statistical relationship between two variables in which an increase in one variable is associated with an increase in the other variable. There exists a robust positive association between many aspects of the subject under study, such as Aftertaste (0.87), Acidity (0.82), Body (0.90), Balance (0.87), and Overall (0.87). This implies that increased levels of light may result in elevated evaluations for these traits. There is a moderate positive connection observed between the variable Totals and the given coefficient of 0.73. There exists a moderate negative association between the variables Fragrance/Aroma and Flavor, with respective correlation coefficients of -0.49 and -0.12. This suggests that there may be a negative correlation between higher levels of light and the scores given to these traits.

Moreover, the associations between features such as Fragrance/Aroma and Flavor imply the existence of potentially ideal thresholds of temperature, humidity, and light levels that can be manipulated to get desired sensory qualities in the dry process. Based on the correlation matrix supplied, it appears that there is no discernible linear link between the environmental parameters (Temperature, Humidity, Light) and the chemical components (Caffeine(ng), Chlorogenic (ng), Theophylline(ng)) in the context of the dry process. The observed correlations exhibit a tendency towards being low or approaching zero. The variables under consideration in this study are temperature, humidity, and light. There is a lack of significant relationships observed between these chemical components (Caffeine, Chlorogenic acid, Theophylline) and the environmental parameters under consideration. The observed correlations exhibit a spectrum ranging from minimal to moderate strength and lack uniformity across various chemical compounds.

**Caffeine** exhibits a modest inverse relationship with temperature (-0.25) and a little direct relationship with chlorogenic acid (0.55). The observed correlations exhibit a lack of significant strength. The variable **Chlorogenic (ng)** exhibits a moderate positive association (0.43) with the variable Temperature. This indicates that there is a tendency for greater temperatures to be linked with increased levels of Chlorogenic acid. Nevertheless, the correlation observed is not highly robust.**Theophylline** exhibits weak relationships with temperature (0.07) and chlorogenic acid (0.12). The observed correlations exhibit a rather low magnitude. In the context of the dry process, there is limited evidence to suggest significant, direct correlations between the chemical constituents (Caffeine, Chlorogenic acid, Theophylline) and the environmental variables (Temperature, Humidity, Light). This implies that additional variables or interplays might exert a greater influence on the chemical composition of coffee beans in the context of the dry processing method.

#### Honey process

**Fig 9:**
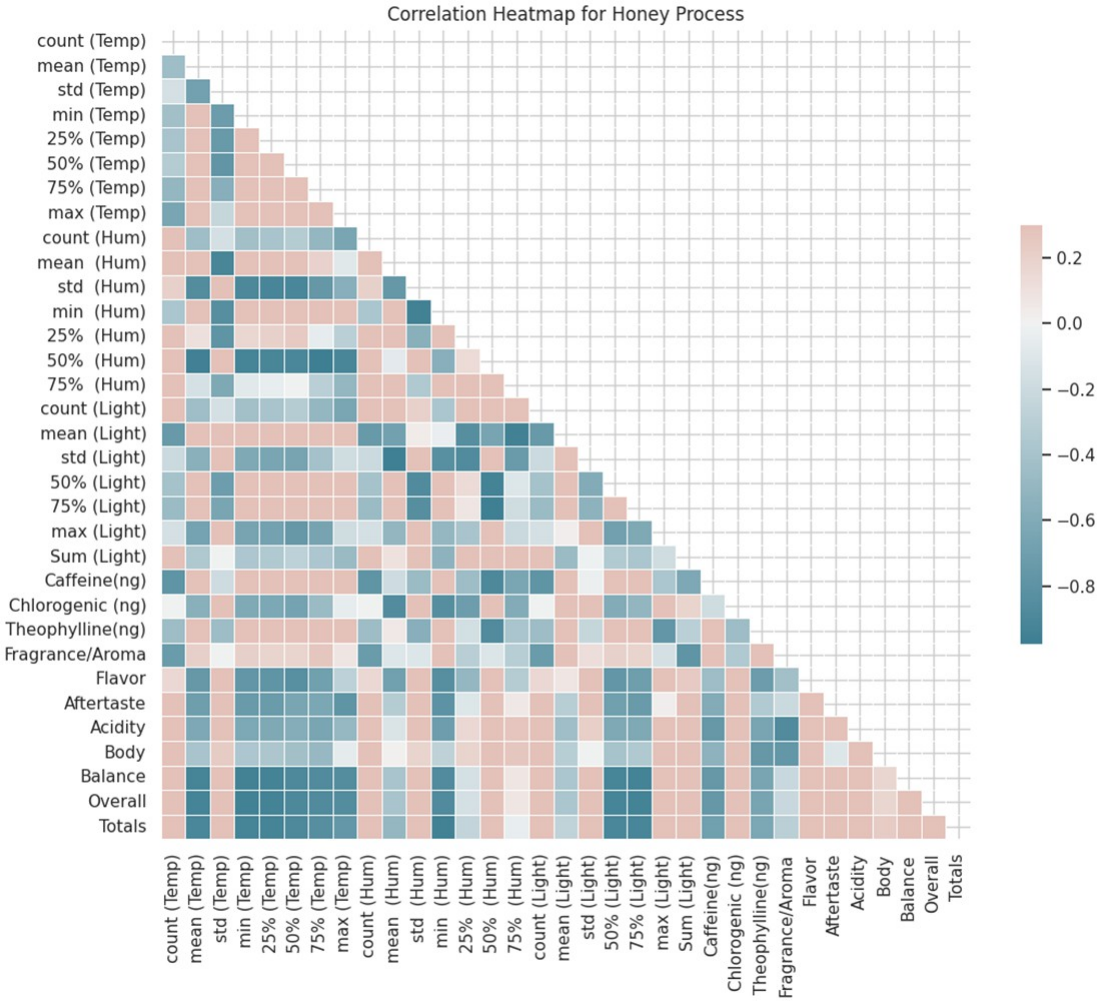
Correlation heatmap for honey process.

To conduct a comprehensive analysis of correlations, the Honey process (as shown in Fig 9) is of paramount importance.

*Temperature:* The average temperature (mean (Temp)) exhibits a significant inverse relationship with the majority of sensory characteristics, such as Fragrance/Aroma (-0.73), Flavor (-0.75), Aftertaste (-0.71), and Acidity (-0.62). This finding indicates a negative correlation between higher temperatures and scores for these qualities. Humidity, also referred to as Hum, is a measure of the amount of water vapor present in the atmosphere.

*Humidity:* The average humidity (mean (Hum)) exhibits a modest positive connection with Fragrance/Aroma (0.21) and Flavor (0.16). This finding indicates a modest positive correlation between humidity and perceived sensory characteristics.

*Light intensity:* The average light intensity (mean (Light)) exhibits a significant inverse relationship with the majority of sensory qualities, akin to temperature. There exists a negative link between the variable in question and the attributes of Fragrance/Aroma (-0.69), Flavor (-0.75), Aftertaste (-0.71), and Acidity (-0.62). This suggests a negative correlation between increasing light intensity and scores for these qualities.

*The chemical constituents:* Caffeine exhibits robust negative associations with Temperature (-0.79), Light (-0.74), and a moderate negative association with Humidity (-0.44). This observation implies a correlation between elevated caffeine levels and reduced temperatures as well as diminished light exposure. The compound chlorogenic (ng) has a slight inverse relationship with temperature (-0.01), a moderate direct relationship with humidity (0.35), and a slight inverse relationship with light (-0.34). Theophylline concentration exhibits a significant positive association with temperature (r = 0.86) and a moderately positive correlation with humidity (r = 0.86). Additionally, there is a weak negative association observed with Light, with a coefficient of -0.45.

**The sensory attributes** refer to the characteristics perceived by the senses, such as taste, smell, touch, sight, and hearing. The sensory qualities, namely Fragrance/Aroma, Flavor, Aftertaste, Acidity, Body, Balance, Overall, and Totals, typically exhibit significant interrelationships. This finding suggests that there is a positive correlation between sensory assessments, which aligns with our expectations. The Overall score has significant negative relationships with Temperature (-0.93) and Light (-0.93), suggesting that elevated temperatures and increased light intensity are linked to diminished overall scores.

In conclusion, the Honey process demonstrates that temperature and light intensity exert significant influences on the chemical composition, particularly caffeine and theophylline, as well as the sensory characteristics of coffee. There exists a negative correlation between elevated temperatures and light intensity and diminished scores in the majority of sensory qualities. The factors of humidity and chlorogenic acid exhibit comparatively lesser associations with the aforementioned variables. It is important to note that the observed interactions are merely correlations and should not be interpreted as implying causality. Therefore, additional experiments and thorough analysis are required to demonstrate any causal relationships.

#### Washed process

**Fig 10:**
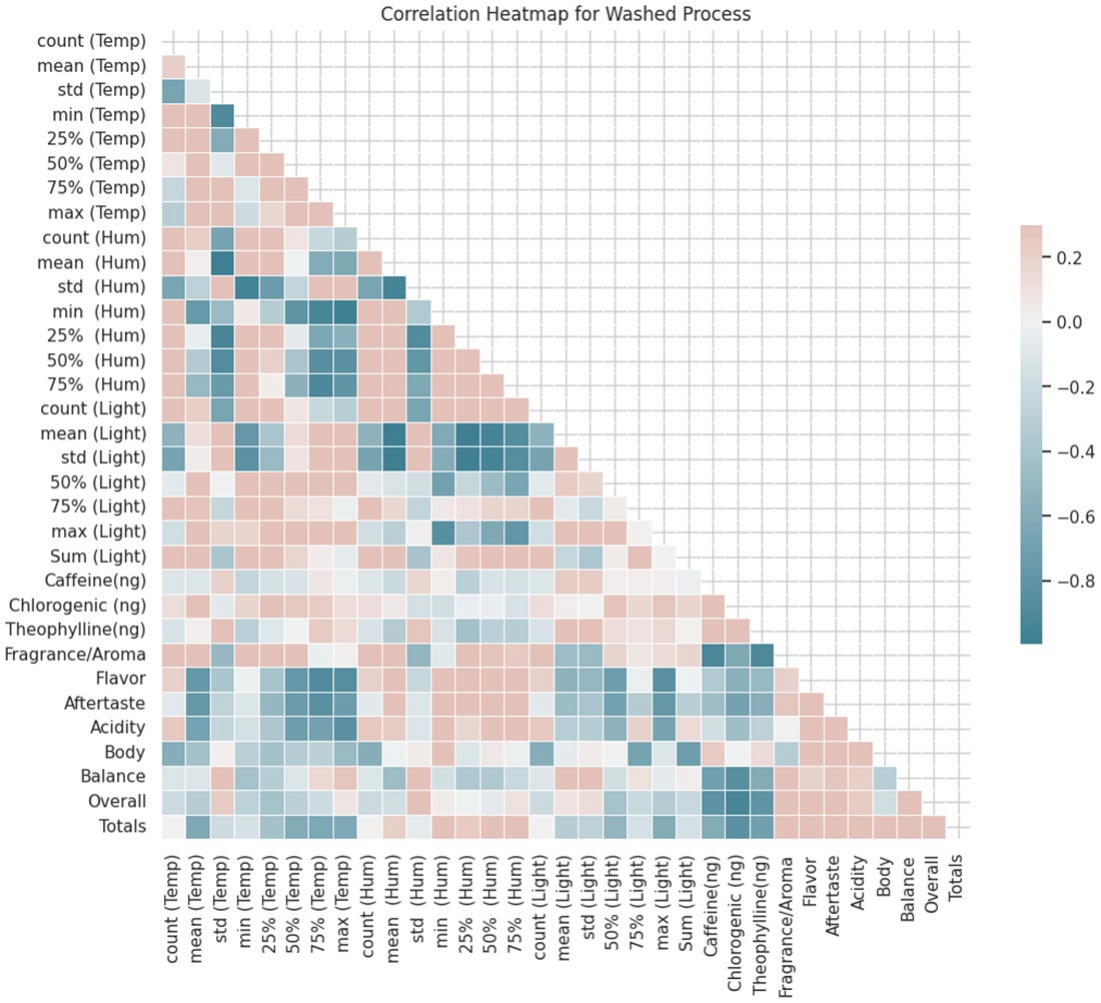
Correlation heatmap for washed process.

Fig 10 presents a heatmap illustrating the correlations among several characteristics or variables associated with the drying process of washed coffee. Every individual cell inside the heatmap illustrates the correlation between two variables, where the color of the cell signifies the magnitude and direction of the association.

**Temperature** There exists a positive association between the variables of count, mean, min, and 25%. This observation implies that there is a positive correlation between temperature and the aforementioned factors, indicating that an increase in temperature is likely to result in an increase in these variables. There exists a negative link between the standard deviation (std) and the values of 50%, 75%, and the maximum (max). This suggests that there is an inverse relationship between temperature and the variables in question, wherein an increase in temperature is associated with a drop in these variables.

*Humidity:* The data demonstrates a robust positive association with count, mean, minimum value, and the 25th percentile. This observation suggests that there is a positive correlation between humidity and the mentioned variables. There is a negative association seen between the standard deviation (std) and the values of 50%, 75%, and the maximum (max). This implies that a rise in humidity is associated with a decrease in these variables.

*Light intensity:* There exists a moderate positive association between the variables of count, mean, and 50%. This suggests that there is a positive correlation between the variables and the intensity of light. There exists a substantial negative association between the standard deviation (std), 75th percentile (75%), and maximum value (max), while a mild negative correlation is observed with the sum. This observation implies that there is an inverse relationship between the growth of these variables and the decrease of light.

*Chemical components (Caffeine, Chlorogenic, Theophylline):* These components typically have low correlations with the majority of other factors. The variables in the dataset exhibit a reasonably high degree of independence from one another.

*Sensory attributes (Fragrance/Aroma, Flavor, Aftertaste, Acidity, Body, Balance, Overall, Totals):* The sensory qualities exhibit limited connections with the majority of other variables. Nevertheless, certain variables exhibit moderate relationships among themselves. As an illustration, various aspects of flavor such as flavor and aftertaste, flavor and overall impression, and so forth, can be considered. This implies that there may be a correlation between specific sensory qualities.

## Discussion

A deep comprehension of the complex relationship between environmental factors and the final coffee quality gave rise to the idea for this paper. The primary goal of our research was to demonstrate how environmental variables such as temperature, humidity, and light intensity shape coffee’s unique characteristics. We set out to fill this knowledge gap after seeing a significant lack of studies that linked environmental factors, chemical components, and sensory qualities in the coffee industry. Our insatiable need to know how different processing techniques affect the subtleties of coffee’s flavor and aroma motivated us to dig further into the topic.

Strengthening industry-wide quality control procedures was the fundamental goal of our research. The goal was to provide stakeholders with useful information derived from a combination of weather tracking, chemical analysis, and sensory evaluations. It’s obvious that chemical compositions and environmental factors have a major impact on the expiry and shelf life of coffee goods. A coffee’s aroma, flavor, and quality are all profoundly influenced by its key chemical components, such as chlorogenic acid, theophylline, and caffeine. Environmental variables like drying time, temperature, humidity, and light exposure significantly affect the stability of these chemicals.

The sensory qualities and shelf life of coffee goods are affected by the degradation of these chemical components when exposed to bad environmental conditions. Prolonged drying times can hasten the breakdown of some compounds, while inadequate drying can cause moisture levels to rise, making it easier for mold and bacteria to grow. Heat has the ability to speed up chemical reactions, which could change flavor profiles or cause volatile aromatic chemicals to evaporate. Because ultraviolet light speeds up chemical interactions, it affects the coffee’s sensory properties. The coffee industry can benefit from a greater understanding of these complex relationships to set stricter quality assurance standards and determine more accurate expiration dates. Manufacturers can maintain the appropriate chemical composition and produce high-quality coffee with a prolonged shelf life by expertly regulating and optimizing these variables.

Our research lays the groundwork for further studies that will hopefully lead to more exact storage recommendations and more accurate expiration date designations. The creation of predictive models that use big data analytics to foretell the degradation of quality depending on many environmental factors can be an undertaking for the future. Investigating eco-friendly packaging and new ways to store coffee could also greatly increase their shelf life, decrease their environmental impact, and make customers pleased.

## Conclusion

This research investigates the intricate relationship among environmental factors, chemical constituents, and sensory attributes within the context of coffee production. The findings indicate that maintaining accurate temperature control is essential for optimizing efficiency during the drying process, as greater temperatures have been found to be associated with elevated drying rates. The effect of humidity in the drying process and the chemical composition of coffee is of utmost importance, as higher levels of humidity result in extended duration of drying. The preservation of aromatic chemicals in coffee beans is also influenced by humidity, which has a direct impact on the fragrance and olfactory characteristics of the final coffee product.

The intensity of light, especially in the context of dry processing, exerts a notable influence since higher levels of light exposure have been found to be associated with enhanced efficiency in the drying process. Nevertheless, an overabundance of exposure has the potential to detrimentally impact the taste and overall excellence of the coffee. In order to optimize drying efficiency, it is advisable for producers to take into account the utilization of both natural and artificial lighting systems.

A chemical study has demonstrated a clear relationship between environmental conditions and the chemical composition of coffee. Specifically, increased temperatures have been found to be associated with higher levels of caffeine, whilst humidity plays a role in regulating the concentration of chlorogenic acid. The provided information holds significant value for coffee farmers as it enables them to enhance their processing conditions for optimal results.

The research also underscores variations in sensory characteristics across various processing methods, underscoring the significance of judiciously selecting the suitable processing strategy to get desired taste features. This information has the potential to assist producers in making well-informed decisions aimed at improving the quality, flavor, and overall excellence of their coffee products. The integration of weather monitoring, chemical analysis, and sensory assessments has the potential to optimize quality control protocols in the coffee industry, thereby ensuring the delivery of exceptional coffee goods to consumers.
